# Early characterization of PSA dynamics in patients with metastatic hormone-sensitive prostate cancer receiving apalutamide-based regimens

**DOI:** 10.3389/fonc.2026.1787398

**Published:** 2026-03-26

**Authors:** Peiyan Yu, Min Gao, Runxuan Li, Tonxin Hu, Ziling Liu

**Affiliations:** Oncology Department of Cancer Center, The First Hospital of Jilin University, Changchun, Jilin, China

**Keywords:** androgen receptor inhibitor, apalutamide, metastatic hormone-sensitive prostate cancer, prostate cancer, real-world evidence

## Abstract

**Objective:**

This single-center, real-world study exploratorily compared early PSA kinetics between apalutamide plus androgen deprivation therapy (ADT) and triplet therapy (ADT, apalutamide, and docetaxel).

**Methods:**

This study was designed as a single-center retrospective cohort study. A total of 36 patients with newly diagnosed metastatic hormone-sensitive prostate cancer (mHSPC) and adverse prognostic features between January 2022 and September 2024 were included. Adverse prognostic features were defined as a Gleason score ≥8 and/or high-volume disease according to the CHAARTED criteria. Patients were stratified according to first-line treatment into a doublet group receiving apalutamide plus ADT (n = 17) and a triplet group receiving docetaxel plus apalutamide and ADT (n = 19). All patients received ADT induction for no more than 2 weeks prior to the initiation of apalutamide.

The primary endpoints were the time from apalutamide initiation to the first achievement of PSA90 and PSA95, defined as a ≥90% and ≥95% decline in PSA from baseline, respectively. Secondary endpoints included time to PSA <0.2 ng/mL, time to undetectable PSA (PSA <0.09 ng/mL), PSA nadir level, and time to nadir, and PSA levels at predefined time points.

Given baseline imbalances in tumor burden and Gleason score between the two groups, multivariable Cox proportional hazards models were applied for adjustment. Restricted sensitivity analyses were conducted to assess the robustness of the findings.

**Results:**

The triplet group had a higher proportion of patients with high-volume disease and higher overall Gleason scores, indicating a greater baseline disease risk.

Time-to-event analyses showed that the median time to PSA90 was 0.9 months in the triplet group compared with 2.1 months in the doublet group. Similarly, the median time to PSA95 was 1.0 months in the triplet group versus 2.1 months in the doublet group. After adjustment for age, Gleason score, CHAARTED tumor volume, and baseline PSA (log10-transformed) in multivariable Cox regression models, the triplet regimen remained independently associated with a shorter time to achieving PSA90 and PSA95 (PSA90: adjusted hazard ratio [aHR] = 2.50, 95% CI 1.16–5.40; PSA95: aHR = 2.22, 95% CI 1.03–4.78).

Regarding secondary endpoints, no statistically significant differences were observed between the two groups in time to PSA <0.2 ng/mL, time to undetectable PSA (PSA <0.09 ng/mL), PSA nadir level, or time to nadir. Likewise, no significant differences were detected in absolute PSA levels at 3, 6, and 12 months after treatment initiation. Restricted sensitivity analyses yielded results consistent in direction with the primary analyses.

**Conclusion:**

In this real-world cohort characterized by high-risk features, although patients in the triplet group had a greater baseline disease burden, the addition of docetaxel was associated with a more rapid and deeper decline in PSA. However, PSA levels at subsequent predefined time points converged between the two groups.

Given the limitations in sample size and follow-up completeness, the findings of this study should be considered exploratory. Whether the triplet regimen confers long-term benefits in hard clinical endpoints and which patient populations are most likely to benefit, requires validation in larger, well-designed prospective studies with standardized follow-up.

## 1 Introduction

Prostate cancer is the second most commonly diagnosed malignancy and the fifth leading cause of cancer-related death among men worldwide (1, 2). With population aging and the widespread use of prostate-specific antigen (PSA) testing, the incidence of prostate cancer in China has shown an increasing trend (3).

Metastatic hormone-sensitive prostate cancer (mHSPC) represents a critical stage in the continuum of disease management. The primary therapeutic goals at this stage are to delay progression to castration-resistant disease while preserving quality of life and improving long-term survival. Clinical studies have demonstrated that more effective disease control during the mHSPC phase may prolong the subsequent therapeutic window after progression to castration-resistant prostate cancer (CRPC). Therefore, optimization of initial treatment strategies is particularly important for patients with high-risk disease (4–6).

Apalutamide, a next-generation androgen receptor (AR) antagonist, inhibits the AR signaling pathway at multiple levels. Based on the findings of the TITAN trial, apalutamide in combination with androgen deprivation therapy (ADT) has been established as a standard first-line treatment for mHSPC, demonstrating significant survival benefits and a reduced risk of disease progression (4). With its widespread adoption in clinical practice, the evidence base has expanded from randomized controlled trials to real-world settings. Several recently published multicenter retrospective cohort studies and international analyses have further confirmed the biochemical control and tolerability of apalutamide across diverse populations, supporting its robust external validity (7–10).

However, despite consistent evidence supporting its overall efficacy, differences in early antitumor activity between the apalutamide-based doublet regimen and the triplet regimen incorporating docetaxel remain unclear in patients with high-risk mHSPC. Although overall survival remains the gold standard endpoint for efficacy assessment, PSA kinetics, as a key biomarker, provides important early risk-stratification value in clinical practice (11). Previous analyses have suggested that achieving a deep PSA response early during treatment is associated with improved long-term survival outcomes. Therefore, elucidating the differences in early PSA kinetics between triplet and doublet regimens is critical for optimizing individualized treatment strategies in patients with high tumor burden.

For patients with mHSPC who present with a high baseline tumor burden or other high-risk features, AR pathway inhibition alone may be insufficient to achieve a rapid reduction in tumor burden in the early phase of treatment. Landmark trials such as CHAARTED and STAMPEDE have demonstrated that the addition of docetaxel to ADT improves survival outcomes in patients with high-volume disease (12). The ARASENS trial further suggested that intensifying treatment with an AR pathway inhibitor on top of docetaxel confers additional survival benefits (13, 14).

However, direct comparative evidence between the triplet regimen of docetaxel plus apalutamide and ADT and the apalutamide plus ADT doublet regimen remains limited. In particular, head-to-head comparisons focusing on early PSA kinetics in real-world settings are scarce (15).

To address this evidence gap, we conducted a single-center retrospective real-world cohort study to exploratorily compare early PSA kinetics between apalutamide-based first-line doublet and triplet regimens. The co-primary endpoints were time to achievement of PSA90 and PSA95. We further assessed time to PSA <0.2 ng/mL, time to undetectable PSA, PSA nadir, PSA levels at predefined time points, and safety profiles. This study aimed to provide additional evidence to inform initial treatment intensification strategies for patients with high-risk mHSPC.

## 2 Methods

### 2.1 Study design and ethics

This study was designed as a single-center retrospective cohort study. Clinical data were collected from the electronic medical record system of the First Hospital of Jilin University. The study protocol was approved by the Medical Ethics Committee of the First Hospital of Jilin University (Approval No. 2025-625). As the study utilized previously collected, de-identified clinical data and did not involve any intervention in patient management, the requirement for informed consent was waived by the ethics committee. The data cutoff date for this analysis was October 1, 2025.

### 2.2 Study population

We consecutively enrolled patients newly diagnosed with mHSPC at our institution between January 2022 and September 2024 and received a first-line apalutamide-containing regimen.

The inclusion criteria were as follows: histologically or cytologically confirmed prostate adenocarcinoma; radiologically confirmed distant metastases; at least one adverse prognostic feature, defined as a Gleason score ≥8 or high-volume disease according to the CHAARTED criteria; receipt of either apalutamide plus ADT or docetaxel plus apalutamide and ADT as first-line therapy; availability of baseline PSA data and at least 12 months of PSA follow-up after treatment initiation, with a follow-up frequency of at least once every 3 months in principle.

The exclusion criteria included: prior systemic antitumor therapy for prostate cancer (ADT induction for no more than 2 weeks before initiation of apalutamide was permitted as castration initiation); pathological evidence of neuroendocrine differentiation or small-cell carcinoma; concomitant active malignancies; treatment discontinuation or loss to follow-up within the 12-month observation period for reasons other than disease progression; missing key baseline variables; or severe comorbidities or major organ dysfunction rendering patients unsuitable for the relevant treatments.

### 2.3 Treatment regimens and grouping

The date of first apalutamide administration was defined as the index date. Patients in the doublet group received apalutamide 240 mg orally once daily in combination with ADT. Patients in the triplet group received docetaxel at a dose of 75 mg/m² every 3 weeks for a planned total of six cycles in addition to apalutamide plus ADT. Short-term ADT induction prior to apalutamide initiation was permitted; however, the induction duration was limited to no more than 2 weeks.

### 2.4 Data collection

Baseline variables collected included age, baseline PSA level, Gleason score, sites of metastasis, and tumor burden status. Baseline PSA was defined as the value measured closest to treatment initiation within 30 days prior to the index date. Outcome-related data included duration of apalutamide treatment, number of docetaxel cycles administered, and serial PSA measurements with corresponding dates during treatment.

Safety data were obtained from outpatient and inpatient medical records, as well as laboratory test results at our institution. For patients who were referred back to other hospitals for continued treatment, only safety information documented during their treatment at our institution was available; adverse events occurring after transfer could not be systematically captured.

### 2.5 Study endpoints

The primary endpoints were time to deep PSA response, defined as the time from the index date to the first achievement of PSA90 and PSA95. PSA90 was defined as a ≥90% decline in PSA from baseline, and PSA95 as a ≥95% decline from baseline. Patients who did not reach the respective thresholds were censored at the date of last follow-up or at initiation of subsequent therapy, whichever occurred first.

Secondary endpoints included time from the index date to the first achievement of PSA <0.2 ng/mL, time to undetectable PSA (PSA <0.09 ng/mL), and absolute PSA levels at 3, 6, and 12 months after treatment initiation. For fixed time-point analyses, a window of ±14 days around the target date was allowed. PSA nadir and time to PSA nadir were also described, with PSA nadir defined as the lowest recorded PSA value within the 12-month observation window. Given the lower limit of detection for some PSA measurements, between-group comparisons of nadir levels were exploratorily analyzed using Tobit regression.

The safety endpoint was treatment-related adverse events, summarized as incidence of grade 1–2 and grade 3–4 events according to the Common Terminology Criteria for Adverse Events (CTCAE). The safety analysis population included patients who had received at least one dose of the respective drug at our institution and for whom safety data were available. The apalutamide safety cohort comprised 77 patients, and the docetaxel safety cohort comprised 37 patients. As some patients were subsequently referred to other institutions for continued treatment in real-world practice, adverse event data after transfer were incomplete; therefore, the reported safety incidence reflects only events documented at our institution.

### 2.6 Statistical analysis

All statistical analyses were performed using SPSS version 26.0 (IBM Corp., Armonk, NY, USA) and R version 4.2.0 (run in RStudio). Continuous variables were presented as mean ± standard deviation (SD) or median with interquartile range (IQR), depending on distributional characteristics. Between-group comparisons were conducted using the independent-samples t test or the Mann–Whitney U test, as appropriate. Categorical variables were summarized as counts and percentages, and compared using the chi-square test or Fisher’s exact test.

Time-to-event outcomes were analyzed using the Kaplan–Meier method to generate survival curves and estimate median times with corresponding 95% confidence intervals (CIs). Between-group differences were assessed using the log-rank test. To evaluate the independent association between treatment regimen and time to PSA90 and PSA95, multivariable Cox proportional hazards regression models were constructed. Covariates included age, Gleason score, CHAARTED tumor volume, and log10-transformed baseline PSA.

Given the lower limit of detection for PSA (0.09 ng/mL), Tobit regression was applied for exploratory comparison of PSA nadir (the lowest PSA value recorded within the observation window), in order to appropriately account for left-censored data and reduce potential bias from simple substitution methods. In the Tobit model, PSA nadir was treated as the dependent variable, treatment group as the primary independent variable, and age, Gleason score, CHAARTED tumor volume, and log10-transformed baseline PSA were included as covariates. Parameters were estimated using maximum likelihood, and results were reported as regression coefficients with 95% CIs.

Given the close association between treatment allocation and tumor burden, a restricted sensitivity analysis was further conducted, including only patients with high-volume disease was conducted to assess the consistency of the findings. No *a priori* sample size calculation was performed, and inferential tests were used for exploratory purposes. As PSA90 and PSA95 were co-primary and highly correlated endpoints, no adjustment for multiple comparisons was applied. All statistical tests were two-sided, and a P value <0.05 was considered statistically significant.

Safety analyses were descriptive in nature, and no inferential comparisons between treatment groups were performed.

## 3 Results

### 3.1 Patient screening and enrollment process

A total of 82 patients newly diagnosed with mHSPC between January 2022 and September 2024 were screened. According to the predefined criteria, 46 patients were excluded, and 36 patients were ultimately included in the efficacy analysis, comprising 17 patients in the doublet group and 19 in the triplet group.

**Figure 1:**
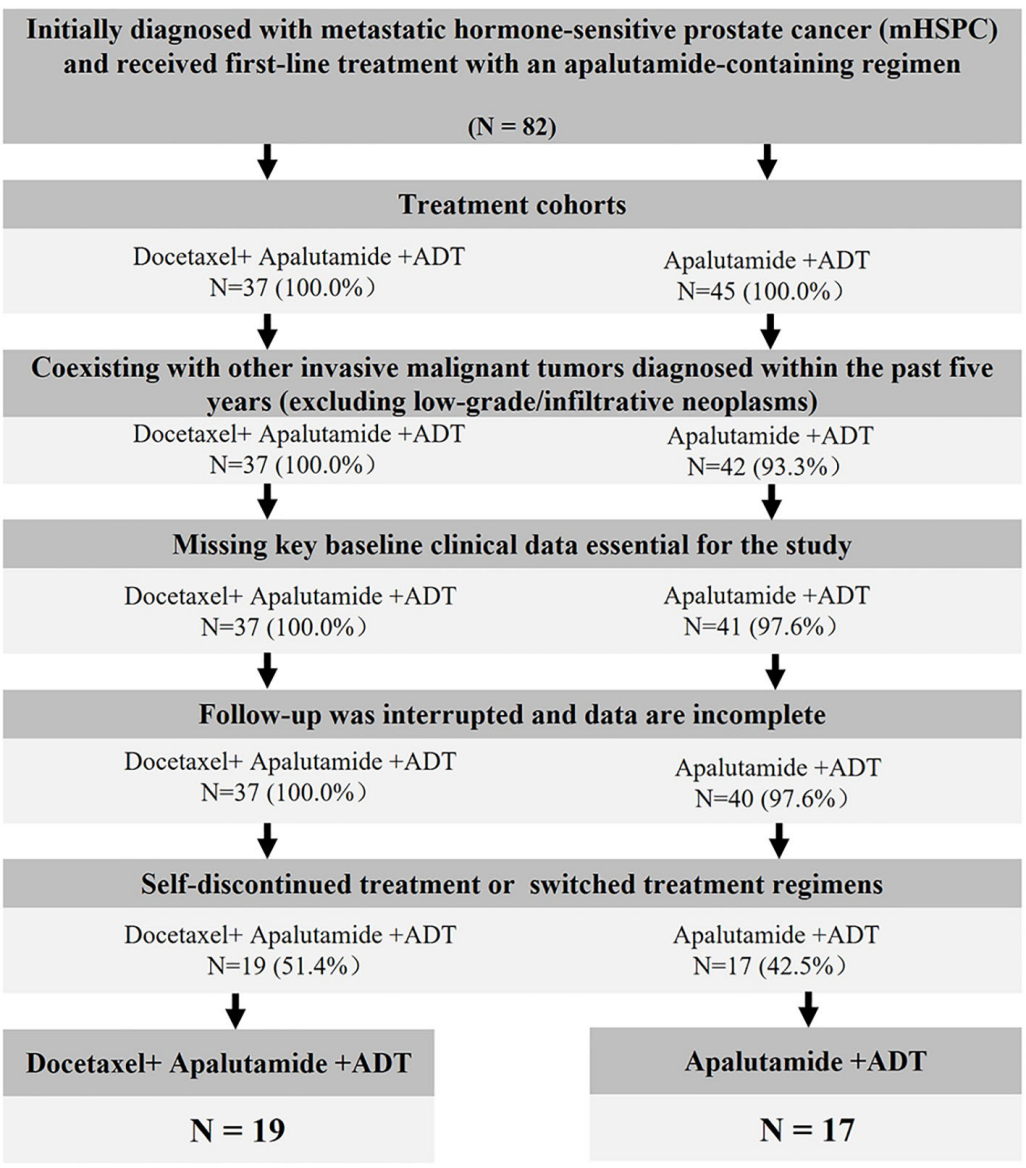
Patient identification flowchart. mHSPC, metastatic hormone-sensitive; ADT, androgen deprivation therapy.

Among the excluded cases, a relatively high proportion discontinued treatment or switched regimens for reasons other than disease progression during the observation period, including self-discontinuation, transfer to other institutions resulting in interrupted follow-up, and treatment discontinuation or modification due to drug-related adverse events. This pattern suggests the potential presence of selective follow-up and survivor bias. The patient selection process is illustrated in **Figure 1**.

For safety analyses, the apalutamide safety cohort included 77 patients, and the docetaxel safety cohort included 37 patients. As safety analyses did not require 12 months of PSA follow-up, the size of the safety population differed from that of the efficacy analysis cohort.

### 3.2 Baseline characteristics

Baseline demographic and clinicopathological characteristics of the two groups are summarized in **Table 1**. There was no statistically significant difference in age between the two groups (P = 0.312). The duration of ADT induction prior to apalutamide initiation was comparable, and the proportion of patients who received short-term ADT induction did not differ significantly between groups (P = 0.662). Baseline PSA levels (log10-transformed) were also similar between the two groups (P = 0.117).

**Table 1:** Baseline characteristics.

Characteristic	Triplet therapy n=19	Doublet Therapy n=17	P-vaule
Age, mean± SD [Q1, median, Q3]	68.7±4.7 [64.0, 69.0, 72]	71.0± 8.2 [65.5, 71.00, 76.0]	0.312
Gleason score, n (%)			0.014
≤7	0 (0)	1 (5.9)	
8	14 (73.7)	8 (47.1)	
9	2 (10.5)	8 (47.1)	
10	3 (15.8)	0 (0)	
Bone metastasis, n (%)	19 (100)	15 (88.2)	0.216
Visceral metastasis, n (%)	12 (63.2)	3 (17.6)	0.08
CHAARTED High-volume, n (%)	19 (100)	13 (76.5)	0.04
Log10-transformed Baseline PSA, ng/ml, mean±SD [Q1, median, Q3]	2.91± 0.46 [2.77, 2.94,3.24]	2.54±0.89 [1.76, 2.69, 3.24]	0.117
ADT lead-in before apalutamide, n (%)	8.0 (7-12)	2 (11.8%)	0.662
ADT lead-in duration, median, days (range)	8.0 (7-12)	8.5 (7-10)	–

Regarding metastatic sites, bone metastases were common in both groups (P = 0.216). Visceral metastases were more frequent in the triplet group (63.2% vs 17.6%), although this difference did not reach statistical significance in the present sample (P = 0.080). Compared with the doublet group, the triplet group had a significantly higher proportion of patients with high-volume disease as defined by the CHAARTED criteria (100% vs 76.5%, P = 0.040). In addition, the distribution of Gleason scores differed significantly between groups (P = 0.014), indicating an imbalance in baseline disease risk.

Accordingly, subsequent analyses were adjusted using multivariable models, and restricted sensitivity analyses including only patients with high-volume disease were conducted to assess the robustness of the findings.

### 3.3 Primary endpoints

**Figure 2:**
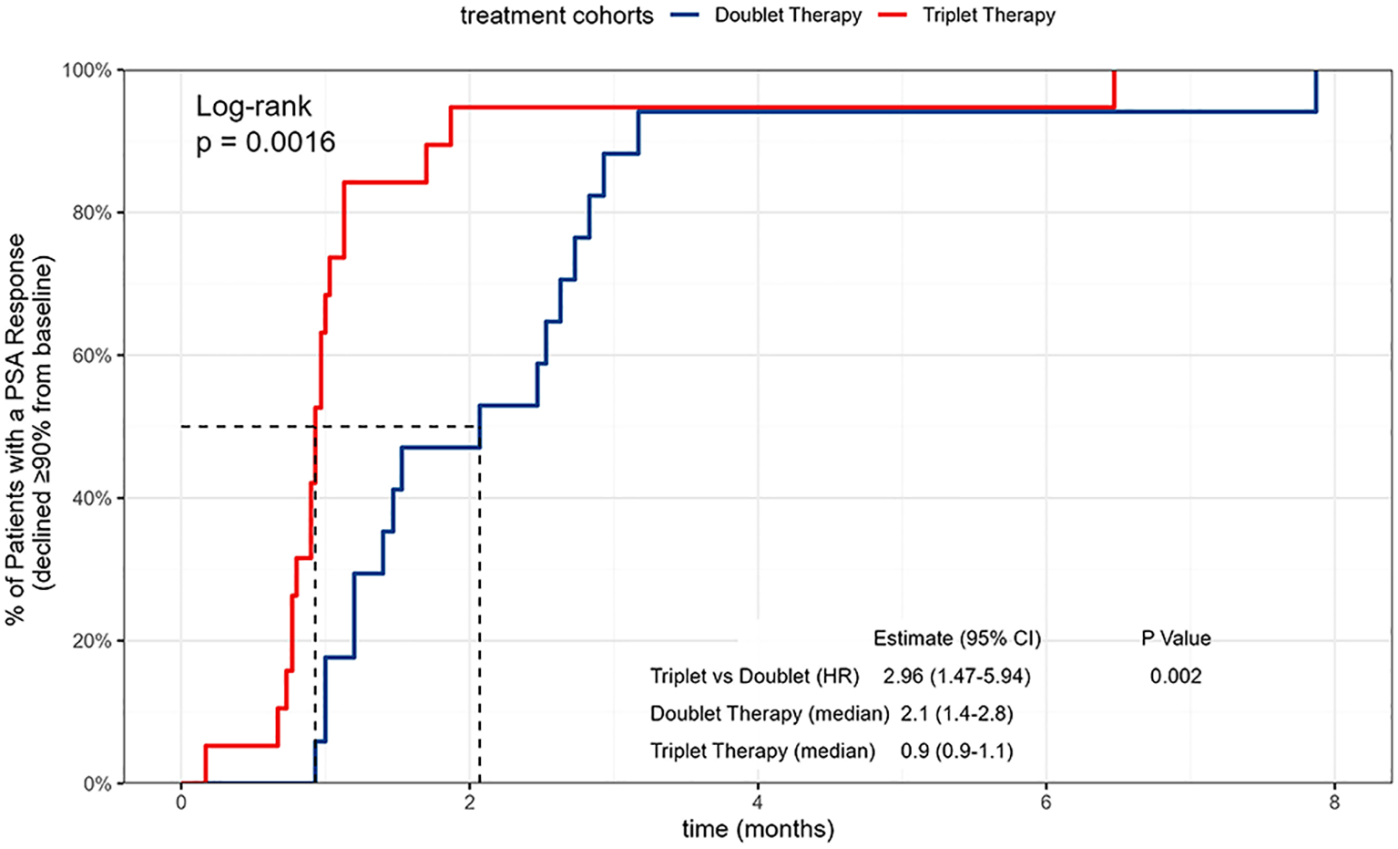
Kaplan-Meier curves of time to PSA90 in patients with metastatic hormone-sensitive prostate cancer (mHSPC) receiving different apalutamide-based regimens.

**Figure 3:**
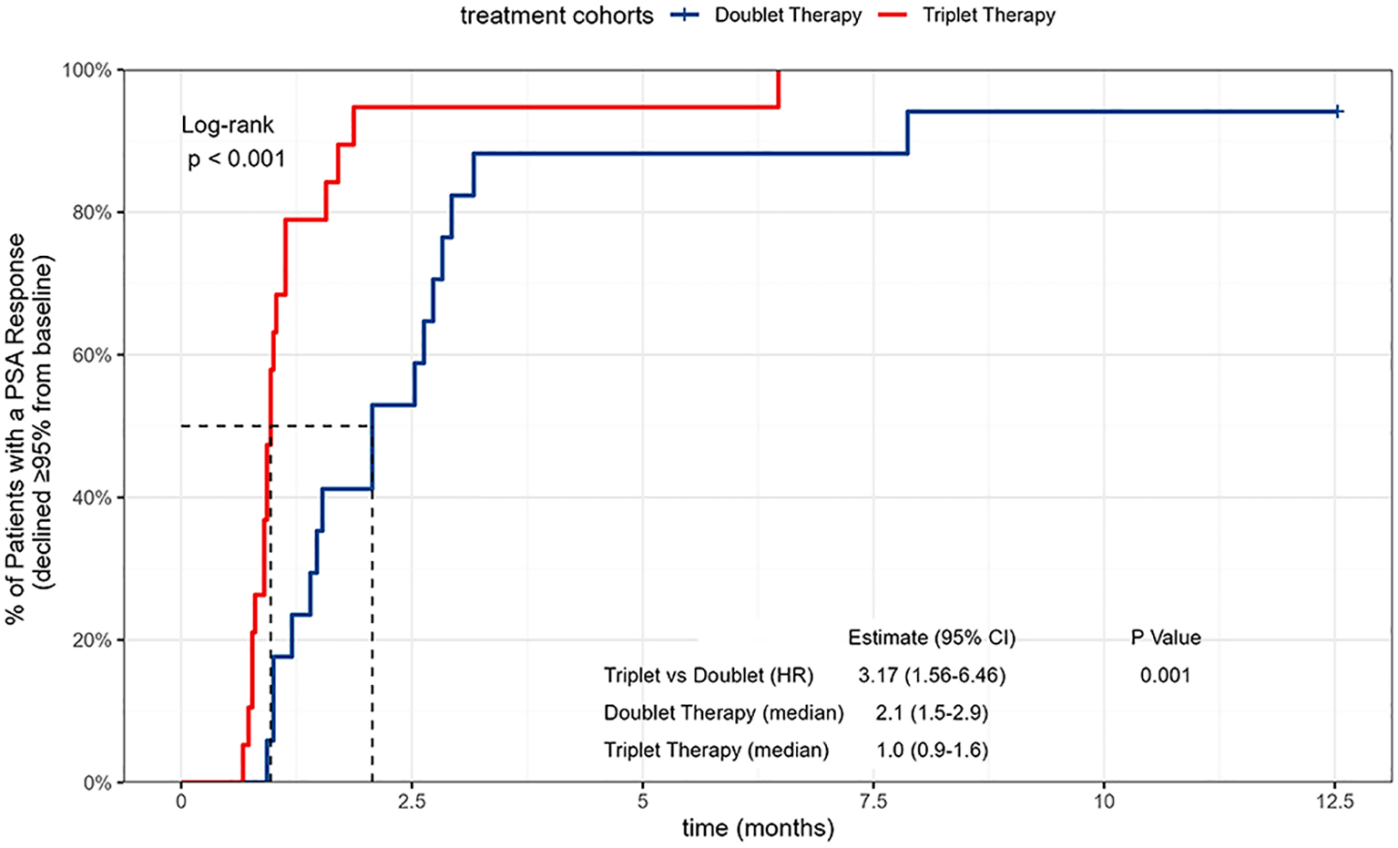
Kaplan-Meier curves of time to PSA95 in patients with metastatic hormone-sensitive prostate cancer (mHSPC) receiving different apalutamide-based regimens.

**Figure 4:**
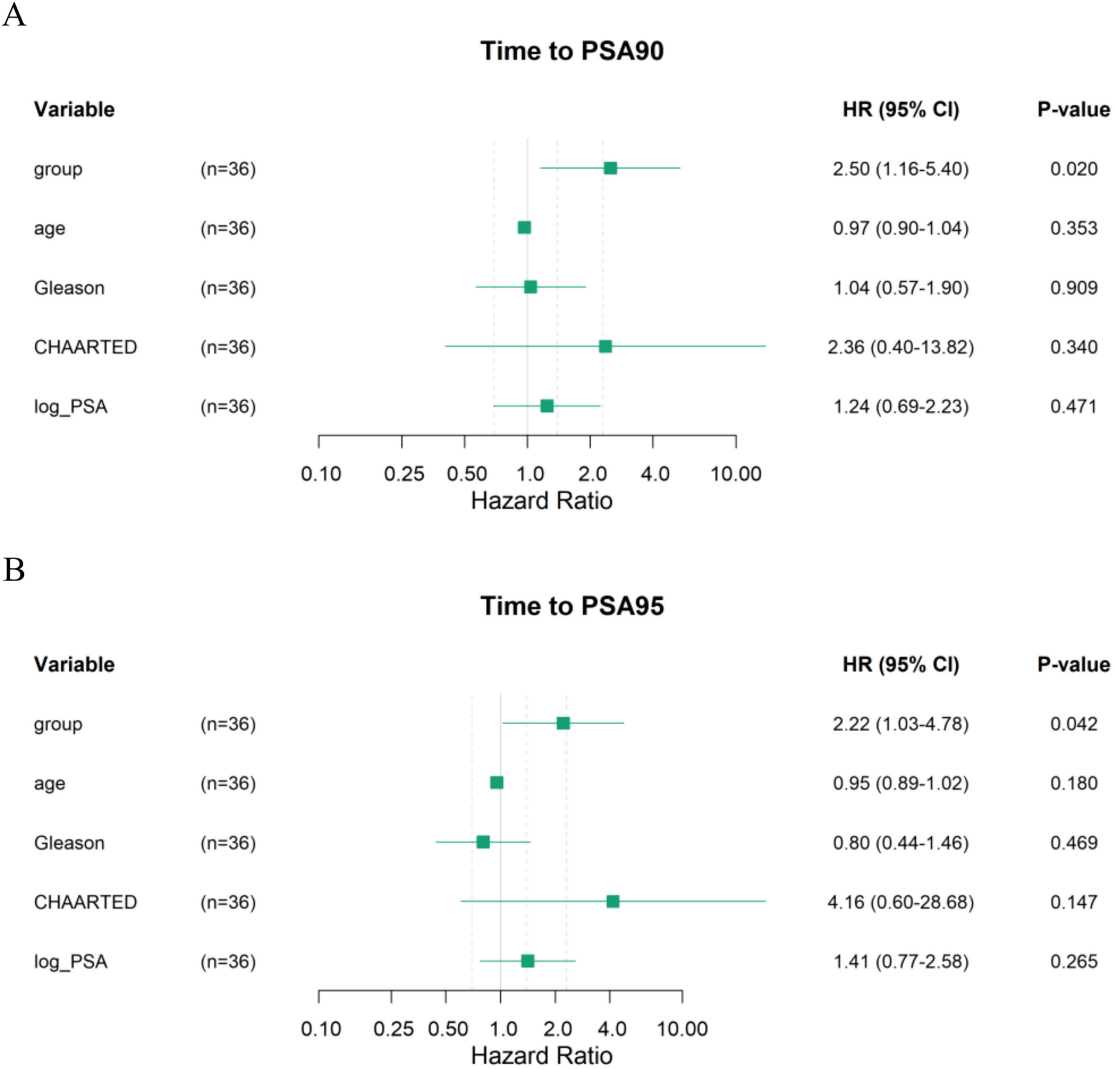
**(A)** Multivariable Cox regression analysis of factors associated with time to PSA90 achievement (aHR and 95% CI). **(B)** Multivariable Cox regression analysis of factors associated with time to PSA95 achievement (aHR and 95% CI).CHAARTED indicates high-volume disease according to the CHAARTED criteria; Log_PSA represents log10-transformed baseline PSA.

All included patients completed at least 12 months of PSA follow-up, with a mean follow-up duration of 17.3 months. Kaplan–Meier analyses showed that the median time to PSA90 was 0.9 months in the triplet group and 2.1 months in the doublet group, with a statistically significant between-group difference (**Figure 2**). The median time to PSA95 was 1.0 months in the triplet group compared with 2.1 months in the doublet group, and this difference was likewise statistically significant (**Figure 3**). In multivariable Cox regression analyses adjusting for age, Gleason score, CHAARTED tumor volume, and log10-transformed baseline PSA, the triplet regimen remained independently associated with a shorter time to achieving PSA90 and PSA95 (PSA90: aHR = 2.50, 95% CI 1.16–5.40, P = 0.020; PSA95: aHR = 2.22, 95% CI 1.03–4.78, P = 0.042) (**Figure 4**).

In the restricted sensitivity analysis including only patients with high-volume disease per the CHAARTED criteria (19 in the triplet group and 13 in the doublet group), Kaplan–Meier analysis demonstrated a shorter median time to PSA90 in the triplet group than in the doublet group (0.9 vs 1.5 months; log-rank P = 0.0018). In the unadjusted Cox model, the triplet regimen was associated with a shorter time to PSA90 (HR = 2.39, 95% CI 1.14–5.00), and this association remained significant after adjustment for covariates (aHR = 2.39, 95% CI 1.09–5.27, P = 0.030) (**Table 2**).

For PSA95, the median time to response was 1.0 months in the triplet group and 2.1 months in the doublet group (log-rank P = 0.021). The unadjusted Cox model yielded an HR of 2.34 (95% CI 1.11–4.93), and the adjusted model showed an aHR of 2.14 (95% CI 0.98–4.66), indicating borderline statistical significance (P = 0.056). Overall, the sensitivity analyses were directionally consistent with the primary analyses (**Table 2**).

**Table 2:** Restricted sensitivity analysis including only patients with high-volume disease as defined by the CHAARTED criteria: time to achievement of PSA90 and PSA95.

Variable	Triplet therapy (n= 19)	Doublet therapy (n= 13)	*P*-value
Time to PSA90			
Median time, month (95% CI)	0.9 (0.9-1.1)	1.5 (1.2-NR^b^)	0.0018^a^
Unadjusted HR^c^ (95% CI)	2.39 (1.14- 5.00)	Ref	0.021
Adjusted HR^c^ (95% CI)	2.39 (1.09-5.27)	Ref	0.030
Time to PSA95			
Median time, month (95% CI)	1.0 (0.9-1.6)	2.1 (1.2-NR^b^)	0.021^a^
Unadjusted HR^c^ (95% CI)	2.34 (1.11- 4.93)	Ref	0.025
Adjusted HR^c^ (95% CI)	2.14 (0.98-4.66)	Ref	0.056

^a^P values were calculated using the log-rank test; ^b^NR indicates not reached; ^c^HR/aHR were estimated using Cox proportional hazards regression models.

### 3.4 Secondary endpoints

Analysis of secondary endpoints showed that the median time to achieving PSA <0.2 ng/mL and undetectable PSA was numerically shorter in the triplet group than in the doublet group; however, these between-group differences did not reach statistical significance.

Fixed time-point analyses demonstrated that although the triplet group exhibited a more rapid early decline in PSA, absolute PSA levels at 3, 6, and 12 months after treatment initiation gradually converged between the two groups, with no statistically significant differences. A summary of the primary and secondary endpoints—including PSA <0.2 ng/mL, undetectable PSA, PSA nadir, and time to nadir—is presented in **Table 3**.

**Table 3:** Summary of primary and secondary outcomes in the overall study cohort.

Variable	Triplet therapy (n= 19)	Doublet therapy (n= 17)	*P*-value
3-month PSA (ng/mL), M (P25, P75)	0.43 (0.17, 1.55)	0.86 (0.62, 4.36)	0.570
6-month PSA (ng/mL), M (P25, P75)	0.13 (0, 0.56)	0.14 (0,0.83)	0.950
12-month PSA (ng/mL), M (P25, P75)	0 (0, 0.86)	0 (0, 0.22)	0.264
Time to PSA90			
Median time, month (95% CI)	0.9 (0.9-1.1)	2.1 (2.1-2.8)	0.0016^a^
Unadjusted HR (95% CI)	2.96 (1.47- 5.94)	Ref	0.002
Adjusted HR (95% CI)	2.50(1.16-5.40)	Ref	0.020
Time to PSA95			
Median time, month (95% CI)	1.0 (0.9-1.6)	2.1 (1.5-2.9)	<0.001^a^
Unadjusted HR (95% CI)	3.17 (1.56- 6.46)	Ref	0.001
Adjusted HR (95% CI)	2.22 (1.03-4.78)	Ref	0.042
Time to PSA < 0.2			
Median time, month (95% CI)	5.00 (3.6-NR)	5.73 (4.1-NR)	0.430^a^
Unadjusted HR (95% CI)	1.38 (0.61-3.08)	Ref	0.438
Adjusted HR (95% CI)	1.51 (0.59-3.84)	Ref	0.390
Time to undetectable PSA			
Median time, month (95% CI)	7.0 (4.5-NR)	8.0 (5.0-NR)	0.720^a^
Unadjusted HR (95% CI)	1.12 (0.54-2.33)	Ref	0.761
Adjusted HR (95% CI)	1.44(0.59-3.49)	Ref	0.426
Time to PSA nadir			
Median time, month (95% CI)	6.5 (4.5-12)	6.0 (5.0-10.0)	0.69^a^
Unadjusted HR (95% CI)	0.87(0.44-1.72)	Ref	0.688
Adjusted HR (95% CI)	1.25 (0.52-3.02)	Ref	0.616
PSA nadir^b^	Coefficient (95%CI)		
	0.83 (-7.36, 9.02)	Ref	0.843

^a^P values were calculated using the log-rank test; b: Tobit regression adjusted for age, Gleason score, CHAARTED tumor volume, and log10-transformed baseline PSA, with treatment group as the primary independent variable.

### 3.5 Safety analysis

In the docetaxel safety cohort, grade 3–4 adverse events were primarily neutropenia, febrile neutropenia, infections, and anemia. In the apalutamide safety cohort, rash and fatigue were the most commonly reported adverse events, with a subset of patients experiencing grade 3–4 rash.

As a substantial proportion of patients were referred to other institutions for continued treatment, adverse events occurring after transfer could not be systematically captured. Therefore, the reported incidence of treatment-related adverse events in this study may underestimate the true occurrence rates. A summary of treatment-related adverse events is presented in **Table 4**.

**Table 4:** Summary of treatment-related adverse events.

Drug	Adverse events	Grade 1–2 n (%)	Grade 3–4 n (%)
Docetaxel	Neutropenia	8 (21.6%)	3 (8.1%)
(N = 37)	Febrile Neutropenia (FN)	0 (0%)	3 (8.1%)
	Infection/Sepsis	0 (0%)	2 (5.4%)
	Anemia	8 (21.6%)	2 (5.4%)
	Thrombocytopenia	6 (16.2%)	1 (2.7%)
	Peripheral Neuropathy	1 (2.7%)	0 (0%)
	Alopecia	7 (18.9%)	0 (0%)
	Nausea/Vomiting	11 (29.7%)	0 (0%)
	Diarrhea	5 (13.5%)	0 (0%)
	Allergic Reactions/ Infusion Reactions	5 (13.5%)	0 (0%)
Apalutamide	Rash	21 (27.3%)	7 (9.1%)
(N = 77)	Fatigue	15 (19.5%)	0 (0%)
	Hypothyroidism	2 (2.6%)	0 (0%)
	Hypertension	1 (1.3%)	0 (0%)

## 4 Discussion

### 4.1 Main findings and clinical implications

This single-center real-world retrospective cohort study systematically evaluated differences in early PSA kinetics between an apalutamide-based first-line doublet regimen and a triplet regimen incorporating docetaxel in patients with high-risk mHSPC.

Our results demonstrated that although the triplet group had a higher proportion of high-volume disease and a distribution skewed toward higher Gleason scores at baseline, the triplet regimen remained independently associated with a shorter time to achieving PSA90 and PSA95. This association persisted after multivariable Cox regression adjusting for age, Gleason score, CHAARTED tumor volume, and baseline PSA, suggesting that the addition of chemotherapy may enhance early antitumor intensity in high-risk patients, manifesting as a more rapid and profound decline in PSA.

At the same time, our findings indicate that the advantage of the triplet regimen was largely confined to the early phase of treatment in terms of the rate of PSA decline. No sustained between-group differences were observed across subsequent multidimensional endpoints. The two groups demonstrated comparable outcomes in time to PSA <0.2 ng/mL, time to undetectable PSA, PSA nadir levels, and absolute PSA values at predefined time points.

Collectively, these results suggest that while the triplet regimen may offer an advantage in accelerating early biochemical response, the apalutamide-based doublet regimen does not appear inferior in maintaining long-term depth of biochemical suppression.

### 4.2 Comparison with and contextualization within previous studies

First-line treatment strategies for mHSPC have evolved from ADT monotherapy to combination approaches incorporating AR pathway inhibitors or chemotherapy, with further exploration of triplet intensification in selected high-risk populations (16). Landmark registration trials such as TITAN and their *post hoc* analyses have established the efficacy of apalutamide; however, real-world evidence in more heterogeneous clinical settings remains limited and warrants further investigation (17).

Several pivotal studies published in 2025 have helped address this evidence gap and provide important external context for interpreting the present findings. A recent large multicenter retrospective cohort study evaluating the effectiveness of apalutamide in routine clinical practice reported biochemical response patterns and tolerability profiles that were highly consistent with the trends observed in our study (11). In addition, real-world analyses from European and international cohorts have demonstrated that apalutamide-based regimens can induce rapid and deep PSA declines across diverse patient populations. Such evidence spanning different ethnicities and healthcare systems enhances the external validity of our findings (10, 18).

Building upon the latest evidence published in 2025, the present study further refines the characterization of PSA kinetics under different treatment intensification strategies. Consistent with recent literature demonstrating the robust PSA control achieved with apalutamide, the doublet group in our cohort likewise exhibited favorable response rates (19, 20).

Regarding the triplet regimen, our findings indicate an additional benefit in terms of response speed, thereby adding to the current understanding of PSA kinetics. Notably, whereas recently published large-scale cohort studies have primarily focused on overall effectiveness outcomes, the present study places greater emphasis on the early and nuanced kinetic changes associated with the combined use of apalutamide and docetaxel. These complementary lines of evidence collectively support the rationale for treatment intensification in high-risk patients in real-world clinical practice.

### 4.3 Potential mechanisms

The more rapid achievement of PSA90 and PSA95 observed with the triplet regimen may be mechanistically attributed to the complementary effects of cytotoxic chemotherapy and AR pathway inhibition. Docetaxel disrupts microtubule dynamics, thereby inhibiting proliferative tumor cells and inducing apoptosis over a relatively short time frame, which may translate into a rapid reduction in tumor burden (21). In parallel, apalutamide exerts potent and sustained blockade of the AR signaling pathway, effectively suppressing the expansion of androgen-dependent clones. The dual antitumor pressure generated during the early phase of treatment is clinically reflected by a steep decline in PSA levels.

On the other hand, PSA kinetics, as a surrogate marker of tumor biological behavior, are substantially influenced by baseline tumor burden and assay lower limits of detection. In this study, multivariable models and Tobit regression were applied to adjust for potential confounding factors to the greatest extent possible. The findings suggest that the kinetic advantage associated with combination therapy was unlikely to be solely attributable to baseline imbalances, but rather reflects a biological effect inherent to the treatment strategy itself.

### 4.4 Clinical implications

For patients with high-risk mHSPC, treatment decision-making requires a careful balance among potential benefits, toxicity burden, and feasibility of implementation (22). Real-world evidence underscores the importance of incorporating individualized patient characteristics and tolerability assessments into clinical decision-making (23).

In light of our findings, the triplet regimen appears to offer distinct value in achieving a rapid biochemical response. This may be particularly relevant for patients with severe symptoms, those requiring urgent disease control, or individuals who may benefit psychologically from a swift reduction in tumor burden. However, both the present study and recent real-world analyses published in 2025 suggest that under more stringent measures of deep response—such as undetectable PSA—and at predefined time-point assessments, the doublet regimen does not appear to be significantly inferior (10).

Therefore, clinicians should avoid relying solely on the speed of early PSA decline as a surrogate for long-term hard clinical outcomes. Instead, treatment selection should comprehensively consider performance status, comorbidity burden, and the patient’s capacity to tolerate chemotherapy-related toxicities. From a safety perspective, the adverse event profile associated with apalutamide in our cohort was consistent with that reported in recent large-scale studies, supporting its manageable safety profile in real-world practice. In contrast, the increased hematologic toxicity observed with the triplet regimen highlights the need for intensified monitoring and proactive toxicity management.

### 4.5 Study limitations

This study has several limitations. First, as a single-center retrospective analysis with a relatively small sample size and non-randomized treatment allocation, it remains susceptible to selection bias and residual confounding despite multivariable adjustment. Second, PSA assessments were performed according to routine clinical practice rather than at prespecified intervals as in prospective trials, which may have affected the precision of kinetic estimates. In addition, due to limited follow-up duration, radiographic progression-free survival and overall survival outcomes were not available for analysis.

Nevertheless, the direction of our findings is consistent with several large-scale, multicenter real-world studies published in 2025, which partially mitigates the inherent limitations of single-center data. Future large-scale prospective studies with longer follow-up are warranted to determine whether rapid early PSA declines translate into durable survival benefits and to further define the patient populations most likely to benefit from triplet intensification strategies.

## 5 Conclusions

In this real-world cohort of patients with high-risk mHSPC, the combination of docetaxel, apalutamide, and ADT was associated with a shorter time to achieving PSA90 and PSA95. However, no significant differences were observed between groups with respect to secondary endpoints, including time to PSA <0.2 ng/mL, time to undetectable PSA, PSA nadir, and absolute PSA levels at predefined time points.

Given the limitations related to sample size, baseline imbalances, follow-up duration, and the absence of hard clinical endpoints, these findings should be interpreted as exploratory and hypothesis-generating.

## Data Availability

The raw data supporting the conclusions of this article will be made available by the authors, without undue reservation.
